# Application of the Random Forest Classifier to Map Irrigated Areas Using Google Earth Engine

**DOI:** 10.3390/rs13050876

**Published:** 2021-02-26

**Authors:** James Magidi, Luxon Nhamo, Sylvester Mpandeli, Tafadzwanashe Mabhaudhi

**Affiliations:** 1 Geomatics Department, Tshwane University of Technology, Pretoria 0001, South Africa; 2 Water Research Commission of South Africa (WRC), Lynnwood Manor, Pretoria 0081, South Africa; 3 Centre for Transformative Agricultural and Food Systems (CTAFS), School of Agricultural, Earth and Environmental Sciences, University of KwaZulu-Natal, Scottsville, Pietermaritzburg 3209, South Africa; 4 School of Environmental Sciences, University of Venda, Thohoyandou 0950, South Africa

**Keywords:** spatial analysis, water management, change detection, vegetation indices, image classification

## Abstract

Improvements in irrigated areas’ classification accuracy are critical to enhance agricultural water management and inform policy and decision-making on irrigation expansion and land use planning. This is particularly relevant in water-scarce regions where there are plans to increase the land under irrigation to enhance food security, yet the actual spatial extent of current irrigation areas is unknown. This study applied a non-parametric machine learning algorithm, the random forest, to process and classify irrigated areas using images acquired by the Landsat and Sentinel satellites, for Mpumalanga Province in Africa. The classification process was automated on a big-data management platform, the Google Earth Engine (GEE), and the *R*-programming was used for post-processing. The normalised difference vegetation index (NDVI) was subsequently used to distinguish between irrigated and rainfed areas during 2018/19 and 2019/20 winter growing seasons. High NDVI values on cultivated land during the dry season are an indication of irrigation. The classification of cultivated areas was for 2020, but 2019 irrigated areas were also classified to assess the impact of the Covid-19 pandemic on agriculture. The comparison in irrigated areas between 2019 and 2020 facilitated an assessment of changes in irrigated areas in smallholder farming areas. The approach enhanced the classification accuracy of irrigated areas using ground-based training samples and very high-resolution images (VHRI) and fusion with existing datasets and the use of expert and local knowledge of the study area. The overall classification accuracy was 88%.

## 1 Introduction

Existing global food and water insecurity challenges are being compounded by increasing temperatures and drought recurrences and increasing demand for water and food due to population growth [1,2]. The need to meet the growing food requirements is intensifying pressure on freshwater resources, which is a cause for concern under climate change [3–6]. The warming climate is exacerbating the challenge of water scarcity, resulting in increased aridity and shifts in agro-ecological zones, affecting crop yields [7]. Irrigation already uses more than 70% of available freshwater resources, on only 18% of cultivated areas globally [8]. Thus, climate change and the increasing demand for food drive the huge increases in irrigated agriculture, changes that require innovative approaches that improve the productive use of water [3,7]. However, strategies on irrigation expansion require accurate information on the spatial extent of irrigated agriculture, which is currently scant [6,9].

Accurate irrigated area maps are critical for informing policy and supporting decision-makers to formulate coherent and transformative strategies with irrigation as an indispensable climate change adaptation strategy [6]. Besides, accurate information on the extent of irrigated areas goes beyond water resources management; its importance cascades into water and food security and assessing the impact of climate change on the agriculture sector [6,10]. However, knowledge on the actual extent and spatial distribution of irrigated areas remains scant, despite the many attempts that have been made to map irrigated areas worldwide [11–13]. Existing irrigated area datasets developed at varying spatial scales and resolutions, including the FAO (Food and Agriculture Organization of the United Nations) database [14], the Global Map of Irrigated Areas version 5 (GMIA 5.0) [15], the MIRCA2000 product [16], and IWMI’s (International Water Management Institute) irrigated area map [17]. In 2016, IWMI further developed an improved irrigated area map for Asia and Africa using canonical correlation analysis and time-lagged regression at 250 m spatial resolution for 2000 and 2010 [13]. Although these datasets are important for indicating irrigated areas, they generally over-estimate the areas due to the low spatial resolution data used [18]. A recent study in South Africa has shown how low spatial resolution data can result in over-estimates of irrigated areas, particularly in smallholder fields that are about 2 ha in size, very small to be detected by low spatial resolution satellites [18].

Advances in remote sensing technologies in conjunction with the emergence of big data and cloud-based processing platforms such as Google Earth Engine (GEE) are facilitating the classification of irrigated areas within improved accuracies in a time and cost-effective manner, enhancing the monitoring of these at both local and global scales [19,20]. This is aided by freely available high-resolution remotely sensed products and novel non-parametric machine learning algorithms for land use classification [18,21,22]. Supervised image classification machine learning algorithms include Support Vector Machine (SVM), Random Forest (RF), decision tree algorithms, and Extreme Gradient Boosting (XGboost) [23]. Managing the increasingly large volumes of remotely sensed data and products is challenging by traditional processing techniques [9]. These cumbersome and slow pre-processing, processing, and post-processing traditional approaches are being replaced by novel machine learning algorithms embedded in cloud computing big data platforms such as GEE [9,20]. The GEE provides access to many petabytes of remotely sensed spatial datasets, enabling their timely geo-processing using machine learning algorithms and batch processing [9].

Methods that improve the accuracy of irrigated areas are critical for supporting decision-making on irrigation expansion. Advances in machine learning and big data management and processing platforms are improving the accuracy and speed of generating cultivated areas. This study used a non-parametric machine learning algorithm, the random forest, to classify near accurate irrigated areas using high-resolution satellite images of Mpumalanga Province, South Africa. The objective was to develop a spatial model that assists policy and decision-makers to make informed assessments on crop water requirements, water allocation, agricultural land planning, crop evapotranspiration patterns, basin hydrology, and the impact of different types of irrigation at any spatial scale on an annual basis.

## 2 Methods

### 2.1 Description of the Study Area

**Figure 1:**
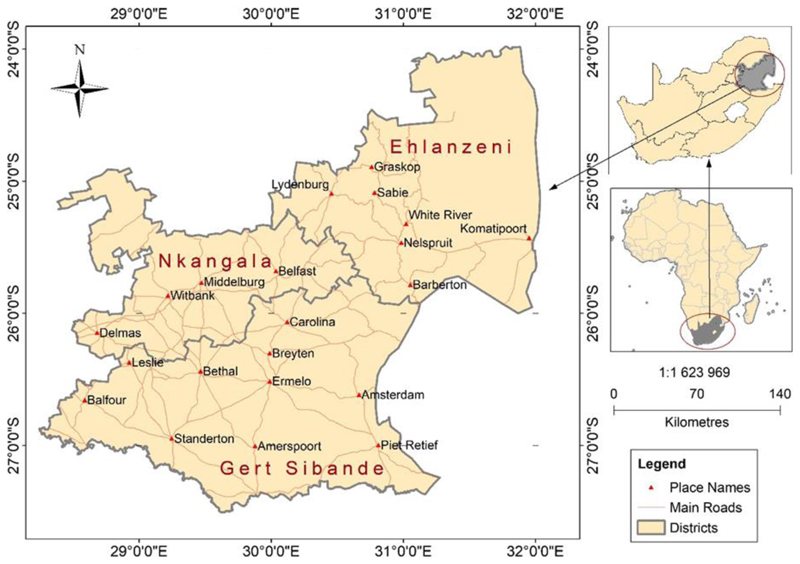
Map of Mpumalanga Province and is location in in South Africa and Africa.

Mpumalanga Province (Figure 1) lies in the east of South Africa, sharing international borders with Eswatini and Mozambique. In South Africa, it shares its borders with the following provinces: Limpopo to the north, Gauteng to the west, the Free State to the southwest, and KwaZulu-Natal to the south. Mpumalanga Province has a total land area of about 76 495km^2^, representing 6.3% of the total land area of South Africa, making it the second-smallest province after Gauteng. It is divided into three district municipalities, namely Ehlanzeni, Nkangala, and Gert Sibande. The topography of the province is characterised by a Great Escarpment, dividing its land area into two major sections: (a) the Plateau area with an elevation of more than 2 000 m to the west, and (b) the Lowveld area in the east [24]. The two topographic zones determine the province’s climate where a temperate climate dominates the Highveld, whilst a sub-tropical climate dominates the Lowveld areas [25].

Vegetation is predominantly grassland. Agriculture is mostly irrigated in both commercial and smallholder farming areas. Smallholder irrigation is predominant in former-homelands, also called Bantustans, areas allocated to indigenous black people during the apartheid era and are generally poorly resourced. A small section in the east lies within the Kruger National Park. The rivers within the province hold some of the most valued ecologically rich systems in the whole of South Africa [26], apart from its rich mineral base that includes huge coal reserves, whose mining is, however, threatening the ecological infrastructure [24].

**Figure 2:**
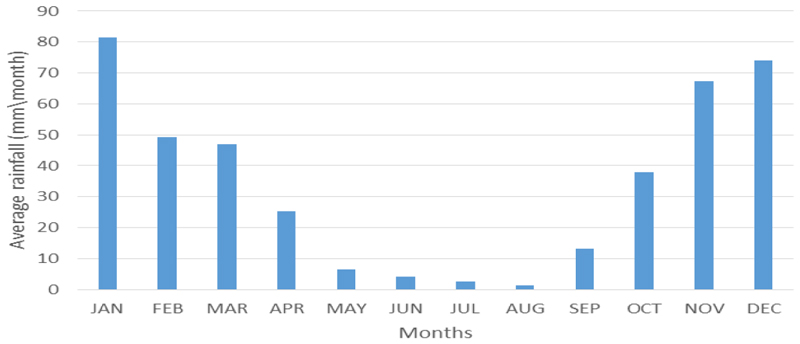
Mean monthly average rainfall of Mpumalanga Province (1972-2018)

Rainfall is seasonal, occurring during the summer season (October to February), as indicated in Figure 2. Like the rest of South Africa, the Mpumalanga Province is water-scarce with almost all its available freshwater resources now allocated, leaving little room for further development [25]. The winter season (April to August) is generally dry and cold (Figure 2), with occasional light snow in the southwestern divide. The average annual temperature is about 20°C, and the mean annual rainfall ranges from 400 mm to 1 500 mm [24].

### 2.2 Methodological Framework

**Figure 3:**
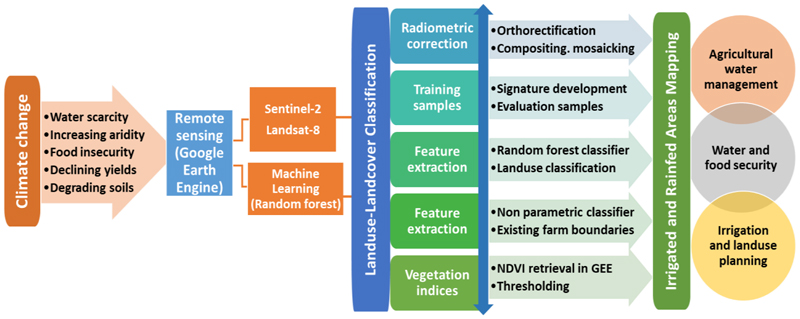
Methodological framework to classify irrigated areas using a non-parametric machine learning algorithm, random forest.

The methodological framework (Figure 3) describes the processes followed in classifying irrigated and rainfed areas in Mpumalanga Province. The study was informed by the increasing warming climate and the intensity of drought, which are compounding water and food insecurity, increased aridity, declining yields, and land degradation [3]. The challenges require informed land use planning and irrigated area development by accurately mapping land use. The Normalised Difference Vegetation Index (NDVI) was used to distinguish irrigated from rainfed areas and compare changes in irrigated areas during 2018/19 and 2019/20 winter growing seasons. The procedure to classify irrigated areas followed four main stages that include (i) data collection, image correction, and pre-processing, (ii) feature extraction, (iii) data integration, and (iv) use of vegetation indices to separate irrigated areas from the rest of cultivated areas (Figure 3). The step-by-step procedure included the mosaicking and compositing of images, signature development and evaluation of samples, use of the random classifier to map cultivated areas, accuracy assessment, and lastly, the use of vegetation indices to separate irrigated areas from other cultivated areas, respectively. The premise was to classify both irrigated and rainfed areas separately, improve the mapping accuracy as an initial step to enhance irrigation and agricultural land use planning, improve agricultural water management and ensure water and food security (Figure 3).

The classification of cultivated areas and the seasonal comparisons in NDVI variations was done using the GEE (see Supplementary Information 1), a big data and cloud computing platform, on cloud-free Sentinel-2 and Landsat-8 images [27]. Satellite images from the Sentinel from July to August for 2020 were mosaicked using GEE. Training sites that were digitised included water, natural vegetation, cultivated land, and built-up areas. The classification was realised through a non-parametric machine learning algorithm, the random forest classifier. The random forest was preferred due to its flexibility and capability to produce, even without hyper-parameter tuning, near accurate land use classifications and is applicable for both classification and regression tasks [28,29]. The classification accuracy was enhanced by integrating existing land use datasets. Average monthly NDVI values were calculated from June to October, a dry season in the study area for 2019 and 2020.

### 2.3 Data Sources

Various time-series and static input data were used for the random forest (RF) classification model on the GEE computing platform. The static input variables refer to existing datasets, terrain, geographic location, and dynamic inputs represent data derived from remote sensing such as NDVI and satellite images and climate data. The premise was to classify cultivated lands at a provincial scale and separate irrigated areas during the dry season that extends from June to October. Data were either obtained from or uploaded to the GEE cloud computing platform for the classification procedure [27].

High-resolution satellite images from the Sentinel 2 sensor (20 m spatial resolution) and the 30 m resolution Landsat images for 2019 and 2020 were accessed from the GEE cloud computing platform (https://developers.google.com/earth-engine/datasets/catalog/). Monthly NDVI data were accessed from Landsat images, also in GEE. Existing agricultural fields and land use datasets were obtained from the Department of Agriculture, Land Reform, and Rural Development’s (DALRRD) Crop Estimates Consortium (CEC) [30] and GeoTerraImage dataset [31], respectively.

### 2.4 The Random Forest Algorithm

The random forest classifier is an ensemble of classification methods consisting of several decision tree models and is expressed as [32]:

(1) $$\left\{DT(x,\theta_{k})\right\}\overset{T}{\underset{k=1}{\;}}$$

where *x* is the input vector, and *θ*_*k*_ denotes a random vector, which is sampled independently but with the same distribution as the previous *θ*_*k*,_ …, *θ*_*k*-1_. *T* bootstrap samples are initially derived from the training data. A no-pruned classification and regression tree (CART) is drawn from each bootstrap sample β where only one of *M* randomly selected features is chosen for the split at each node of CART [32].

The random forest is more robust after minor changes in the input data, and it enhances land use classification accuracy a achieving classifier stability. A *k* number of samples extracted from the training sample set using bootstrap sampling, and the sample size of each sample, are the same as that of the original training set. A *k* number of trees were created for *k* samples, and a *k* number of classification results were obtained. According to the classification results, each record was classified to determine its final land use category. The random forest classifier improves the classification accuracy through its object-based processing algorithms, and its use in remote sensing provides the following advantages:

- It enhances the accuracy of land use/cover mapping as compared to other popular similar algorithms [33]. It maintains the classification error balance when the class size distribution is unbalanced [34].
- The random forest classifier requires little or no manual intervention as it determines data characteristics by itself, simplifying its design process [35].
- Although the random forest algorithm provides various data characterisations, it generally has a quick processing speed [36].

#### 2.4.1 Landcover Classes and Reference Data

Landcover categories were grouped into four types to separate cultivated areas from the rest of the land uses. These included cultivated land, forest, grassland, water, built-up area, and bare land (Table 1). A random sampling method was used to balance the number of training points. Therefore, the number of training points for each landcover class was proportional to the total pixels using the random forest classifier. Existing datasets were used to train and validate the landcover classifications. The reference training and testing datasets were gathered from a time series of both Sentinel and Landsat images and visual interpretation using Google Earth images. The training samples were validated using the Department of Agriculture, Land Reform, and Rural Development’s (DALRRD) cropped area dataset [30] and the GeoTerraImage dataset [31]. The classification resulted in the derivation of the cultivated area dataset of the study area.

#### 2.4.2 Crop Phenology Derivation from NDVI

**Figure 4:**
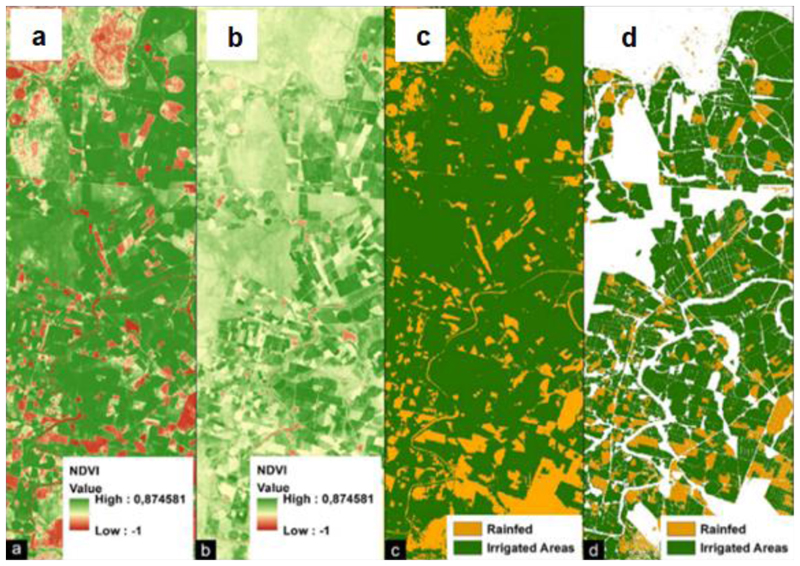
(a) Histogram equalisation of the NDVI average of July 2019; (b) sigmoid stretch of the NDVI average of July 2019; (c) The reclassified NDVI of July 2019 using a threshold; and (d) the classified cultivated land.

Time series data from NDVI was essential for obtaining croplands’ phenological data and for distinguishing irrigated from rainfed areas. Unlike other vegetation types such as grassland and forests, croplands have unique characteristics in the stages of emergence, vegetative growth, senescence, and harvest [37]. These features enable the separation of croplands from other vegetation types using a time series of satellite images (see Supplementary Information 1). NDVI time-series data derived from Landsat images, compiled using Google Earth Engine, were then used to classify and distinguish irrigated from rainfed areas. This was achieved by applying NDVI thresholds ranging between 0.19 and 0.25 for June, July, August, September, and October. The NDVI thresholds were derived using the histogram equalisation and the sigmoid contrast stretch method to distinguish low and high NDVI vigour (Figure 4). Between June and October, it does not rain in the study area, and rainfed agriculture is entirely absent. Therefore, the presence of healthy and green crops signifies the presence of irrigation [38]. Thus, vegetation indices’ seasonal pattern provided the foundation for separating irrigated from rainfed areas through time-series NDVI data.

Histogram equalisation (Figure 4a) and sigmoid contrast stretch (Figure 4b) were used to establish the NDVI thresholds [9,11] used to distinguish vegetated (cropped) and non-vegetated (non-cropped) areas (Figure 4c) from where cultivated lands were derived (Figure 3d). NDVI thresholds ranged between 0.19 and 0.25, and they also confirmed the results of previous studies [38,39]. Cell statistics was applied to combine the reclassified NDVI values (vegetated and non-vegetated) from where the cultivated areas were derived and, ultimately, the separation of irrigated from rainfed areas.

### 2.5 NDVI Trend Analysis over Cultivated Land

**Figure 5:**
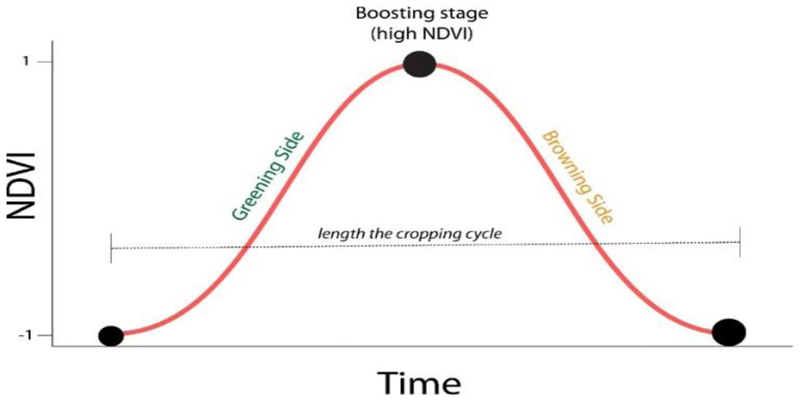
An example of single growing season and related phenological measures analysed through NDVI.

Of note is that outliers and seasonality typically characterise a time series of NDVI data in regions characterised by dry and wet season [40]. The single modal annual rainfall pattern of the study area shows that rainfall (or agricultural season) starts in October and ends in April, indicating a time lag between rainfall and crop phenophases [41] (Figure 5).

As rainfall is a crucial factor in the seasonal crop cycle, there is a positive correlation between rainfall and the seasonal crop growth pattern [42]. Thus, vegetation growth and greening begin in October/November, and browning begins in May/June (Figure 5). This process also applies to rainfed crops in areas with a single modal crop growth cycle. In irrigated areas, greening starts at any time of the dry period from June to October. Croplands could be at a greening stage, yet others are at the boosting stage, while some are at the browning stage (Figure 5).

In most cases, and due to farming relying on rainfall during the wet season, the rainfed crop will start greening in November and browning in May. The greening of irrigated crops begins around June, and the browning in October (Figure 5). Thus, the classification of irrigated areas was assessed from June to October, which coincides with the dry season.

### 2.6 Distinguishing Irrigated from Rainfed Areas

Healthy vegetation reflects more infrared and absorbs more red and blue portions of the electromagnetic spectrum. The red and near-infrared bands are important for calculating NDVI and other vegetation indices as the blue portion is affected by atmospheric scattering [43]. In areas with a single modal annual rainfall pattern, there is less absorption of the visible light and low reflection of the infrared light during the dry season (Figure 5), resulting in low NDVI values. High NDVI values on croplands during the dry season signify irrigation as there is generally no rainfall. NDVI values drop around May to June but picks-up between July and September (Figure 5). Thus, the NDVI threshold ranging between 0.19 and 0.25 was used to isolate irrigated from non-irrigated areas between July and August during the dry season.

## 3 Results and Discussion

### 3.1 Cultivated Areas of Mpumalanga Province

**Figure 6:**
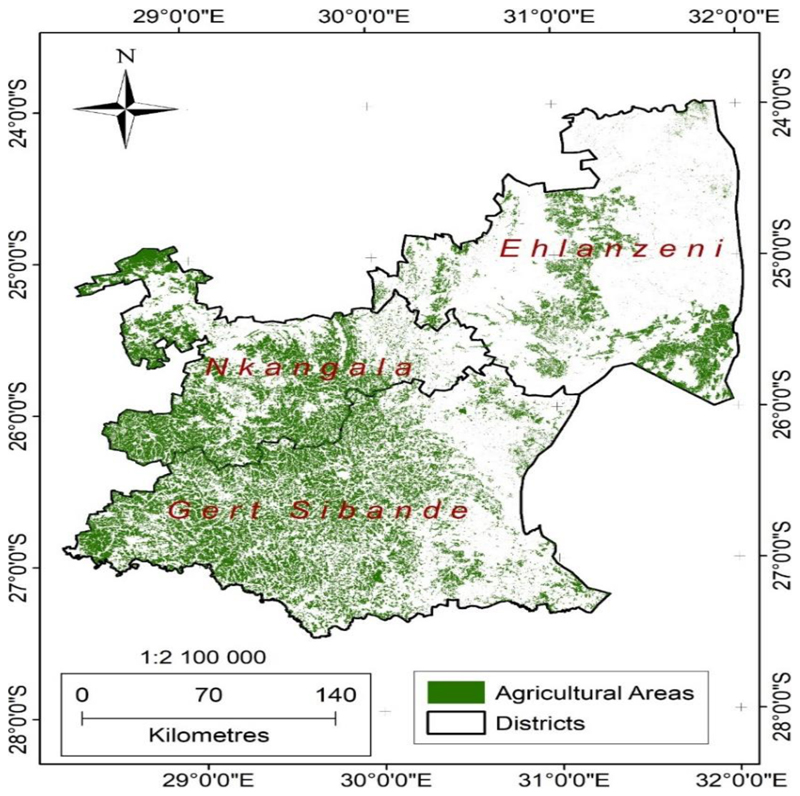
Cultivated lands of Mpumalanga Province derived using the random forest classifier.

The initial product developed from the remote sensing procedure was a dataset of cultivated areas (Figure 6) incorporating both irrigated and rainfed areas for 2020. Twenty-seven percent of the total land areas of Mpumalanga Province is used for agriculture (Figure 6), highlighting the importance of agriculture in the province.

The distribution of cultivated land is uneven as determined by topography, distribution of the river network and soil types, and the location of former homelands. The highest concentration of cultivated land is mainly in the Lowveld area in former homelands, mainly in Nkangala and Gert Sibande districts municipalities. The third district, Ehlanzeni, has limited agriculture activity as most of its area lies within the Kruger National Park and other conservancies.

### 3.2 Accuracy Assessment

**Figure 7:**
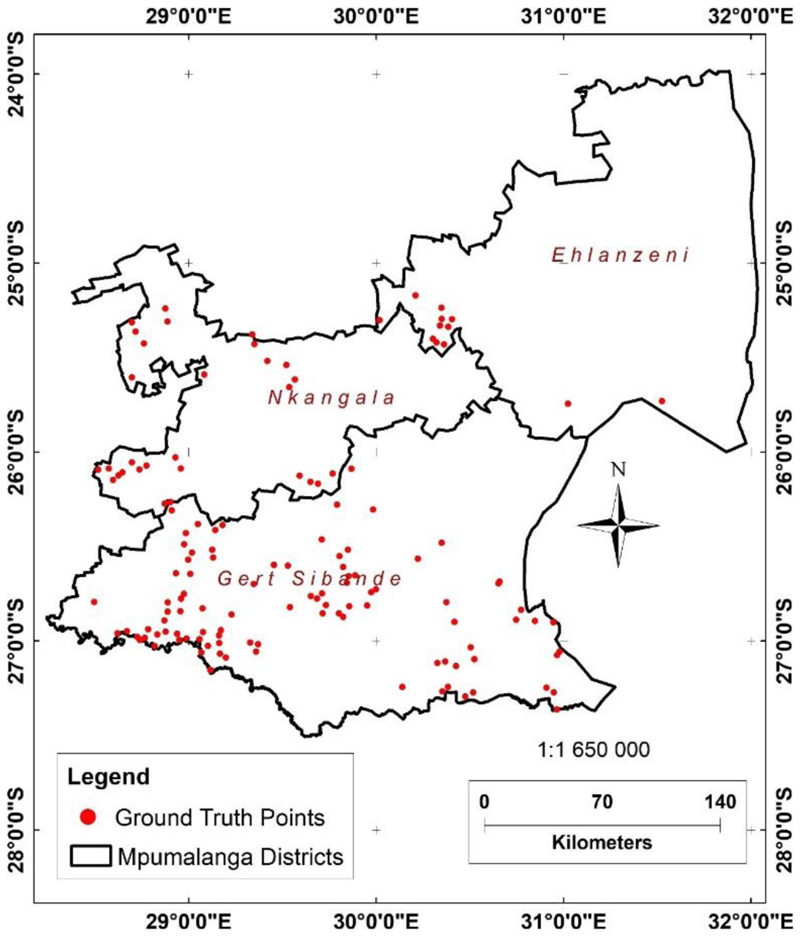
Distribution of accuracy assessment validation points

Accuracy assessment of the mapped cultivated areas was verified by comparing the developed dataset with 130 ground truth points derived from high-resolution Google Earth images (Figure 7). This was enhanced by combining the ground-truth points generated from Google Earth with those from existing datasets using the accuracy assessment tool in ArcMap. This visual interpretation, coupled with the expert and prior knowledge of the study area’s features, contributed to the accuracy assessment using the kappa assessment coefficient algorithm. The ground-truth points were randomly chosen. The accuracy assessment was essential for determining the quality of the classified cultivated area derived through the random forest classifier in remote sensing. The classified cultivated areas’ accuracy was 88%, with a kappa coefficient of 80%, which indicates a very good mapping accuracy. Although relatively high, accuracy can still be improved through post-classification enhancement methods using drones [22].

### 3.3 Separating Irrigated from Rainfed Areas

**Figure 8:**
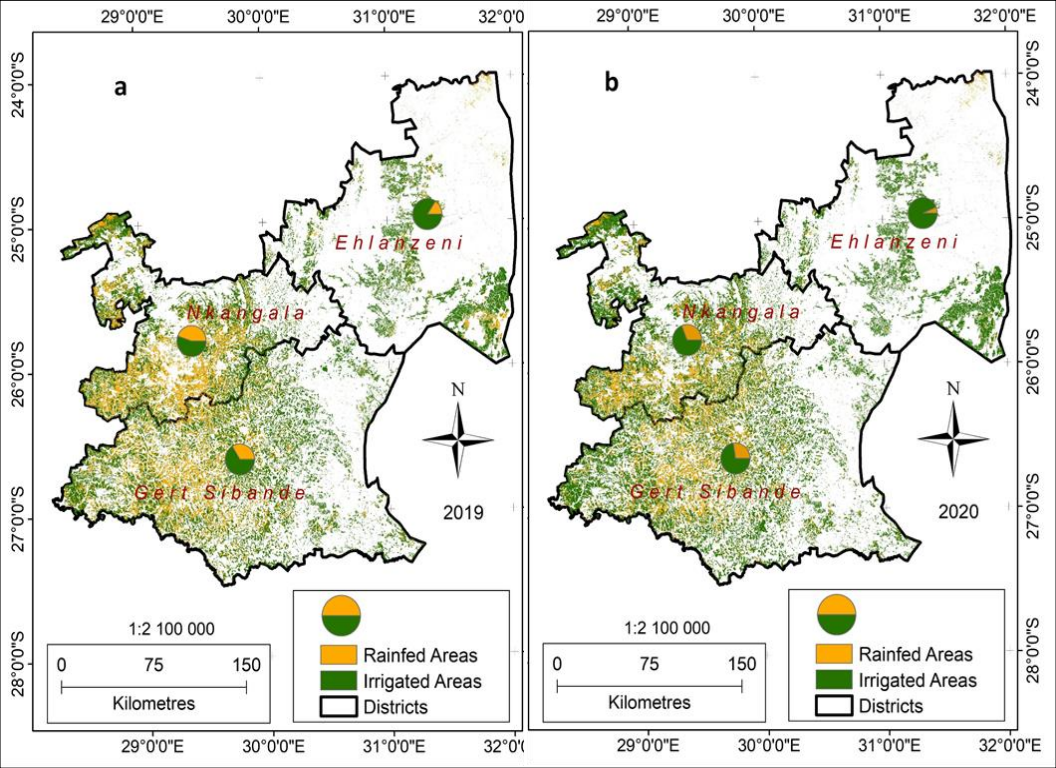
Distribution of cultivated lands in Mpumalanga Province and the proportion between irrigated and rainfed areas per district as well as the changes that took place between 2019 (map a) and 2020 (map b).

The map presented in Figure 8 shows the irrigated and rainfed areas in Mpumalanga, giving the proportion of the two cultivated systems per district municipality. At 75% of the total cultivated area, irrigated land is predominant in the province, where the rainfed area accounts for less than 25% of the total cultivated area. The pie-charts (Figure 8) confirm that the land under irrigation is predominant in the water management area (with an overall proportion of over 75% of the cultivated area), a scenario also given in Table 2. The irrigated area’s predominance also confirms the allocation of most of the available fresh-water resources towards irrigation. The situation also confirms the observations from other studies, which also found out that the province’s available freshwater resources are almost fully allocated [25,26].

**Figure 9:**
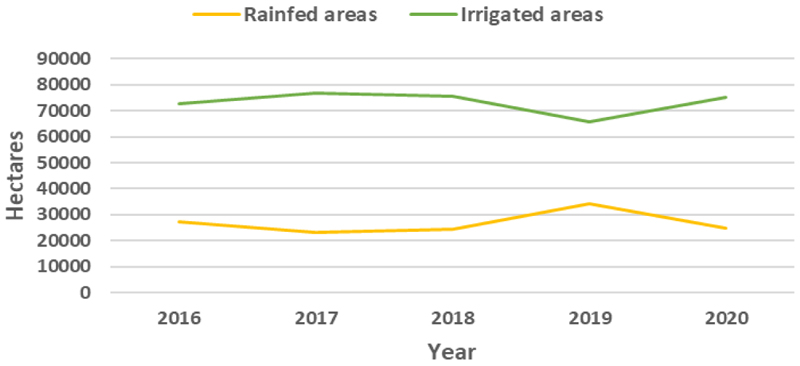
Trends in the irrigated area versus rainfed area in in Mpumalanga Province from 2016 to 2020.

Over time, irrigation trends in Mpumalanga Province indicate that the irrigated area is bigger than rainfed areas, an indicator of high water use in the agriculture sector (Figure 9). Therefore, most of the cultivated land in the province is irrigated as most agricultural land is commercial [44]. The rainfed area has been continuously declining since 2016, except in 2018 when it increased as there was limited irrigation due to drought [3]. However, it significantly declined in 2019 at a time when irrigated further increased (Figure 9). These differences between agricultural types between 2019 and 2020 are noticeable in the maps shown in Figure 8, where it is noticeable that in 2020 most of the cultivated land was irrigated, as indicated by the predominantly green colour.

Irrigation expansion in the province is likely due to ongoing government efforts to equip such areas with irrigation potential and target smallholder farming areas to enhance food security and achieve sustainability by 2030 [45]. However, of concern is that irrigation in smallholder farming areas is usually exempt from water use licences as the water withdrawals are assumed to be negligible [46]. However, this study’s results highlight the need to monitor irrigation expansion at all scales regardless of users, and plan in a more sustainable manner.

### 3.4 Changes in the Irrigated Area between 2019 and 2020

Significant increases are notable in irrigated areas in Mpumalanga Province between the 2019 and 2020 winter growing seasons, a characteristic evident in all districts of the province (Figure 8). The increase only shows that some cultivated areas that were not irrigated in 2019 were irrigated in 2020, not necessarily a change in land area under irrigation. As the rainfed area is less than irrigated in the province, still, between 2019 and 2020, the area that continued as rainfed further decreased by 36.53%. During the same period, the land under irrigation increased by over 12.17% (Table 3), putting a further strain on already scarce water resources [25]. The increase in irrigated area conforms with the National Development Plan (NDP), which promotes the doubling of irrigated areas by 2030 [45], but without considering the availability of other important sectors linked to irrigation like water and energy availability. The land area under irrigation increased during the 2020 Covid-19 pandemic lockdowns when it was expected to decrease. The main contributing factor to the gains in the irrigated area in 2020 is mainly due to agriculture being considered as an essential sector that was allowed to continue operating during the height of the Covid-19 pandemic lockdowns. With many people having lost their other income sources during the lockdown, agriculture became an alternative source of income. In South Africa, there is already irrigation infrastructure that is not being fully utilised [46,47].

Whilst increasing the land under irrigation is necessary to ensure food and water security, there is a need for holistic approaches to ensure that the scarce water resources are equitably distributed with other sectors. Like the rest of South Africa, Mpumalanga Province is water-scarce, and its available freshwater resources are almost fully allocated, leaving little room for future development [25]. Therefore, there is a need for transformative approaches to address resource management in an integrated manner.

The continued decrease in rainfed agricultural area is problematic for policy and decision-makers as increasing the land under irrigation is not always the best-fit solution. Given the worsening water scarcity in South Africa, the reality is that there is a limit to expand the land under irrigation [3,12,48]. A significant part of the solution must be addressed by improving crop water productivity and optimising rainfed systems [49]. Thus, the challenge requires integrated and cross-sectoral approaches in irrigation expansion strategies, translating irrigation into climate change adaptation and mitigation. Previous studies have highlighted the importance of addressing multi-sector and intertwined challenges holistically and avoid the challenges of transferring problems to other sectors [10,50–52]. This calls for transformative approaches such as nexus planning, circular economy, sustainable agricultural systems, and scenario planning in agricultural development [53,54]. For example, this noble initiative to increase irrigation area should also consider water and energy availability. Uninformed irrigation development will only compound the existing water, energy, and energy insecurity [55]. To inform policy and decision-making, there is always a need to have accurate knowledge of the irrigated area’s spatial extent from where water productivity and crop water requirements can be modelled with improved accuracy.

### 3.5 Pros and cons of Using the Random Forest in Land Use Classification

There are more advantages than disadvantages to using the random forests algorithm in land use classification, although there is still room for further improvements. This non-parametric land use classification method offers a more superior method for working with missing data. It substitutes missing values with a variable appearing the most in a particular node [56]. However, in our case, the most important advantage of using the random forest classifier has been the improvement in the mapping accuracy. Furthermore, integrating the random forest technique with big data management platforms facilitated the processing of large data sets with numerous variables within a short space of time. Thus, the technique automatically balances data sets when a class is more infrequent than other classes in the data. It can handle variables fast, making it suitable for complicated tasks [36]. In addition to these advantages, some of the benefits for using the random forest algorithm include (i) the ease with which to interpret the rules using a tree algorithm, (ii) as a non-parametric model, it is easy to add a range of numeric or categorized data layers, (iii) the unimodal training data distribution type is not a prerequisite, and (iv) the classification process is time and cost-effective as long as the programming rules are wellset [29,57].

Although the algorithm has been shown to improve the mapping accuracy and to handle large datasets by integrating with big data management platforms like the GEE, its major disadvantage is that it tends to over-fit the training data, which was the main reason for not providing even better accuracy. This is generally evident when the decision-tree grows too large, with the terminal nodes representing very small subsets of the training data [57]. However, this drawback is overcome by a technique called “pruning”, which simplifies the tree by removing terminal nodes that could be linked to noise in the data [57].

## 4 Conclusions

Advances in remote sensing technologies, alongside the development of machine learning classification algorithms, have facilitated cost and time effective delineation of more accurate irrigated areas. The choice of the most appropriate image classification algorithm is determined by the objectives that need to be achieved; however, the random forest machine learning algorithm used in this study is applicable at any scale and is time and cost-efficiency. This was enhanced using the big-data management platform of the Google Earth Engine (GEE). The high spatial resolution (20 m resolution) cultivated land dataset was developed using a two-year analysis (2019 and 2020), 10-day Sentinel, and 16-day Landsat data. The random forest algorithm handled large volumes of remotely sensed products and reference training and validation datasets from various sources courtesy of the big-data management and processing capabilities of the GEE cloud-computing platform. The combination of methods and approaches in GEE facilitated the rapid classification of more accurate irrigated areas with petabyte volume big-data. The developed, cultivated areas dataset has an overall accuracy of over 88%. The enhanced outputs of the irrigated area mapping are essential for policy and decision-makers to assess vast and complex irrigation systems’ performance in detail. They are critical for accurate monitoring of irrigation activities from the field to transboundary or national scales.

## Supplementary Material

Supplementary material

## Figures and Tables

**Table 1:** Land-cover classification types that were used as training samples.

Land classification	Description
Cultivated land	Land planted with crops, newly opened cropped areas, fallow land
Natural vegetation	Shrublands, forested lands, grasslands, natural or planted forests
Water	All water bodies, including rivers, wetlands, reservoirs, etc
Built-up area	All settlements, including industrial areas

**Table 2:** Disaggregated proportions of cultivated areas per cultivation type between 2019 and 2020.

District	Area (ha)	Rainfed area (ha)	Irrigated area (ha)	Cultivated area (ha)	Rainfed area (%)	Irrigated ar- eas (%)	Cultivated area (%)
**2020**							
Gert Sibande	3 189 239.14	284 963.35	756 614.12	1 041 577.47	27.36	72.64	32.66
Nkangala	1 679 938.90	201 492.75	445 151.50	646 644.25	31.16	68.84	38.49
Ehlanzeni	2 789 686.33	22 592.30	326 176.48	348 768.78	6.48	93.52	12.50
Mpumalanga	7 658 864.36	509 048.40	1 527 942.10	2 036 990.50	24.99	75.01	26.60
**2019**							
Gert Sibande	3 189 239.14	352 240.02	689 337.45	1 041 577.47	33.82	66.18	32.66
Nkangala	1 679 938.90	288 002.67	358 641.59	646 644.25	44.54	55.46	38.49
Ehlanzeni	2 789 686.33	54 785.44	293 983.34	348 768.78	15.71	84.29	12.50
Mpumalanga	7 658 864.36	695 028.12	1 341 962.38	2 036 990.50	34.12	65.88	26.60

**Table 3:** Percentage change in the cultivated areas between 2019 and 2020. Negative changes represent a decrease and positive increases.

	2019	2020	% change
Rainfed areas (ha)	695 028.12	509 048.40	-36.53
Irrigated areas (ha)	1 341 962.38	1 527 942.10	12.176
Total cultivated areas (ha)	2 036 990.50	2 036 990.50	

## Data Availability

Publicly available datasets were analysed in this study. This data can be found here: [https://developers.google.com/earth-engine/datasets/catalog/].
