# Supplementary material for: Application of the Random Forest Classifier to Map Irrigated Areas Using Google Earth Engine
Source: Remote Sens (Basel). Author manuscript; Available in PMC 2024 Jul 19. (PMC7616265; doi:10.3390/rs13050876)
Supplement: Supplementary material [file EMS197514-supplement-Supplementary_material.pdf]

Supplementary Information 1: Code S1

Code 1: Classification of land use using Sentinel

```
Import

var aoi: polygon, 4 vertices //study area
var Water: FeatureCollection (31 elements) //water training sites
var NaturalVegetation: FeatureCollection (490 elements) // Natural Vegetation training sites
var Agrriculture: FeatureCollection (354 elements) //Agriculture Training Sites
var Urban: FeatureCollection (69 elements) //Urban Training sites

var Sentinel2 = ee.ImageCollection('COPERNICUS/S2') // getting the sentinel 2 data
            .filter(ee.Filter.lt("CLOUDY_PIXEL_PERCENTAGE", 10)) // filtering cloud
             .filterDate('2020-07-10' ,'2020-08-31') //filtering dates
             .filterBounds(aoi) // filter based on the geometry

print(Sentinel2) //

//Mosaicking the image and clipping to the region of interest
var sent2Mosaic = Sentinel2.median()
var sent2MosaicClipped = sent2Mosaic.clip(geometry).divide(10000) //

// visualising the mosaicked image false colour compositing
Map.addLayer(sent2MosaicClipped, {bands: ['B12', 'B7', 'B5'], min: 0, max: 1, gamma: 1.5}, 'Sentinel_2 Mosaick')

//Export the mosaicked image.
 Export.image.toDrive({
 image: sent2MosaicClipped.select('B1','B2','B3','B4','B5','B6','B7','B8','B9','B11','B12'),
 description: 'Sentinel2_Mupamalanga,
 scale: 20,
 maxPixels: 1e9,
 region: geometry
});

//Training sites for image classificatin
var newfc = Water.merge(NaturalVegetation).merge(Agriculture).merge(Urban);
print(newfc);

//creating training data
var bands = ['B1','B2','B3','B4','B5','B6','B7','B8','B9','B11','B12'];
var training = sent1MosaicClipped.select(bands).sampleRegions({
   collection: newfc,
   properties: ['landcover'],
   scale:20
   });
print(training);

// Filtering the null property values.
var trainingNoNulls = training.filter(
  ee.Filter.notNull(training.first().propertyNames())
);

//Train the classifier
var classifier = ee.Classifier.smileRandomForest(10).train({
```

```
  features: trainingNoNulls,
  classProperty: 'landcover',
  inputProperties:bands
});
print(classifier);


//Map.centerObject(geometry)

//RunClassification
var classified = sent1MosaicClipped.select(bands).classify(classifier);

// Define a palette for the Land Use classification.
print(classified)
var palette = ['0E469A','004620', '80BB18', 'C18B34'];

// Display the classification result and the input image.
Map.addLayer(classified, {min: 1, max: 4, palette: palette}, 'classified sentinel');

//Exporting the classified image
Export.image.toDrive({
 image:classified,
 description: 'classified_sentinel',
 scale: 20,
 maxPixels: 1e9,
region: geometry
});
```

<u>Code 2:</u> Extracting monthly average NDVI

```
Import
//study area
var aoi: polygon, 4 Vertices
// data
var dataset = ee.ImageCollection('LANDSAT/LC08/C01/T1_8DAY_NDVI')
                  .filterDate('2013-10-01', '2013-10-31');
                                .select('NDVI');
print(dataset)
var ndvi = dataset.reduce(ee.Reducer.mean());
print(ndvi)

//clipping to the study areas
var ndvi_crop = ndvi.clip(aoi);

//visualising the ndvi dataset
var Vis = {
  min: 0.0,
  max: 1.0,
  palette: [
    'FFFFFF', 'CE7E45', 'DF923D', 'F1B555', 'FCD163', '99B718', '74A901',
    '66A000', '529400', '3E8601', '207401', '056201', '004C00', '023B01',
    '012E01', '011D01', '011301'
  ],
};
Map.setCenter(32.5447, -26.00,7);
```

```
Map.addLayer(ndvi_crop, Vis, 'NDVI_col');
//Exporting the data to use in the
Export.image.toDrive({
  image:ndvi_crop,
  description: 'NDVI_10_2013_MP',
  scale:30,
  maxPixels: 1e13,
});
//Further processing like thresholding, cliping were done outside GEE environment
```
